# Suppression of Very Early Stage Of Adipogenesis by Baicalein, a Plant-Derived Flavonoid through Reduced Akt-C/EBPα-GLUT4 Signaling-Mediated Glucose Uptake in 3T3-L1 Adipocytes

**DOI:** 10.1371/journal.pone.0163640

**Published:** 2016-09-26

**Authors:** Yukari Nakao, Hideto Yoshihara, Ko Fujimori

**Affiliations:** Laboratory of Biodefense and Regulation, Osaka University of Pharmaceutical Sciences, 4-20-1 Nasahara, Takatsuki, Osaka, 569–1094, Japan; Brown University Warren Alpert Medical School, UNITED STATES

## Abstract

Baicalein has been used as a Chinese medicine, and is an abundant plant flavonoid present in fruits and vegetables. Here, we examined the effects of baicalein in adipogenesis and investigated its molecular mechanism in adipocytes. Baicalein lowered the intracellular lipid accumulation and decreased the transcription levels of the adipocyte-specific genes in mouse 3T3-L1 adipocytes. Glucose uptake mediated by glucose transporter 4 (GLUT4) was reduced, causing down-regulation of the intracellular lipid accumulation. These reductions were also observed even when baicalein was added in only early stage of adipogenesis (0–2 days) of 6-day-adipogenesis. Chromatin immunoprecipitation assay showed that baicalein decreased the binding level of C/EBPα protein to the promoter region of the GLUT4 gene. Phosphorylation of Akt at 1 h after the initiation of adipogenesis was inhibited by the treatment with baicalein. Inhibition during only the first 1.5 h after the initiation of adipogenesis by baicalein or an Akt inhibitor was enough to decrease the lipid contents in the cells undergoing adipocyte differentiation for 6 days. These results indicate that baicalein decreased the intracellular lipid accumulation by down-regulation of glucose uptake via repression of Akt-C/EBPα-GLUT4 signaling in the very early stage of adipogenesis of 3T3-L1 adipocytes.

## Introduction

Nowadays, an increase in the number of obese subjects has been observed in developed and developing countries [1]. Obesity arises from an imbalance between energy intake and expenditure, and is defined as an abnormal increase in body fat. Obesity is considered to be a risk factor for various metabolic diseases such as hypertension, type 2 diabetes, cancer, and coronary heart disease [2]. The control of obesity is critical in the clinical field. However, adipogenesis process is complex, and its regulation includes the alterations in hormone response and gene expression. A number of transcription factors for the regulation of adipogenesis have been identified [3, 4]. CCAAT/enhancer binding proteins (C/EBPs), peroxisome proliferator-activated receptors (PPARs), and sterol regulatory element-binding protein-1 (SREBP-1) play essential roles in the control of adipocyte differentiation [4]. Subsequently, these key transcription factors activate the expression of a variety of adipocyte-specific genes [4]. Potential therapeutic agents that suppress adipogenesis are important in the prevention of the progression of obesity, and the search of these agents with anti-adipogenic properties has been extensive [5].

**Fig 1:**
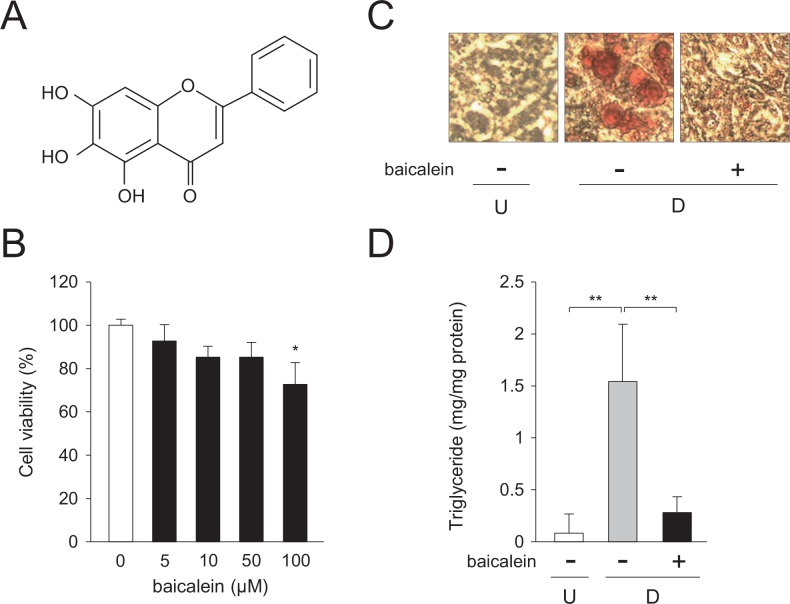
Decrease in intracellular lipid accumulation in 3T3-L1 cells by baicalein. A, Structure of baicalein. B, Cell toxicity of baicalein toward 3T3-L1 cells. Cells were cultured for 6 days in DMEM containing various concentrations of baicalein (0–100 μM). Data are the means ± S.D. *p<0.05, as compared with the vehicle (0 μM). C, Oil Red O staining of baicalein-treated 3T3-L1 cells. Cells (undifferentiated cells: U) were differentiated into adipocytes (differentiated cells: D) for 6 days in DMEM without or with baicalein (0 or 50 μM). Intracellular lipid droplets were stained with Oil Red O. D, Baicalein-mediated suppression of the intracellular triglyceride level of 3T3-L1 cells. Cells (undifferentiated cells: U; *white column*) were differentiated into adipocytes (differentiated cells: D) for 6 days in DMEM without (*gray column*) or without baicalein (50 μM; *black column*). Data are presented as the means ± S.D. **p<0.01, as indicated by the brackets.

It has been reported that natural products lower adipogenesis [6]. Flavonoids are polyphenolic compounds that present in vegetables, fruits, and seeds [7]. They have protective effects against the various diseases [8]. Baicalein (4′,5,7-trihydroxyflavone; Fig 1A) is a plant flavonoid isolated from the roots of *Scutellaria baicalensis* [9]. It is present abundantly in most plants such as fruits and vegetables [10], and it has various biological and physiological activities such as anti-cancer, anti-inflammatory, and antioxidant ones [11, 12]. Although baicalein has a variety of biological activities, the functions of baicalein and its regulatory mechanism in adipose cells remain unclear.

In this study, we found that baicalein lowered the lipid accumulation in mouse 3T3-L1 adipocytes by inhibiting the very early stage of adipogenesis. It did so by decreasing glucose uptake via repression of the Akt-C/EBPα-GLUT4 pathway in adipocytes. Therefore, baicalein has potential for use as an anti-adipogenic agents to reduce the lipid accumulation in adipocytes.

## Materials and Methods

### Materials

Oil Red O, 3-isobutyl-1-methylxanthine (IBMX), dexamethasone, and insulin were purchased from Sigma (St. Louis, MO, USA). Baicalein, 2-deoxy-2-[(7-nitro-2,1,3-benzoxadiazol-4-yl)amino]-D-glucose (2-NBDG), and Akt Inhibitor X were from Cayman Chemical (Ann Arbor, MI, USA). Anti-Akt and anti-phospho-Akt (p-Akt; Thr308) polyclonal antibodies were obtained from Cell Signaling (Danvers, MA, USA). Anti-C/EBPα (C-18), anti-AMPKα (H-300), and anti-phospho-AMPKα (p-AMPK; Thr172) polyclonal antibodies and normal goat IgG were from Santa Cruz Biotech. (Santa Cruz, CA, USA). Anti-β-actin (AC-15) monoclonal antibody was from Sigma. Horseradish peroxidase-conjugated anti-rabbit or anti-mouse IgG antibody was from Santa Cruz Biotech.

### Cell culture

3T3-L1 adipocytes (Health Science Research Resources Bank, HSRRB, Osaka, Japan) were grown in Dulbecco’s modified Eagle’s medium (DMEM) containing 10% (v/v) fetal bovine serum and Penicillin-Streptomycin Mixed Solution (100 U/mL penicillin, 0.1 mg/mL streptomycin; Nacalai Tesque, Kyoto, Japan) as antibiotics. For adipocyte differentiation, 3T3-L1 cells were incubated for 2 days in DMEM containing MDI (0.5 mM IBMX, 1 μM dexamethasone, and 10 μg/ml insulin) and various concentrations of baicalein (0–50 μM). At day 2 after the starting of adipogenesis, the medium was discarded and DMEM with insulin (10 μg/ml) alone was added. The medium was replaced every 2 days. Baicalein was added when the medium was changed except as otherwise stated. Staining of the lipid droplets in the cells with Oil Red O was carried out as described earlier [13].

For the treatment of each differentiation stage, before or after the treatment, the cells were washed with DMEM twice and continued to differentiate into adipocytes for a total of 6 days as described above.

### Cell toxicity assay

3T3-L1 cells were cultured in 96-well plates for 6 days in DMEM with various concentrations of baicalein (0–100 μM). The medium was replaced every 2 days. At the end of the incubation period (at day 6), cell toxic effects of baicalein were evaluated by using Cell Counting Reagent SF (Nacalai Tesque).

### Intracellular triglyceride level

3T3-L1 cells were differentiated into adipose cells for 6 days in DMEM containing baicalein or not. The triglyceride content in the cells as measured at day 6 past differentiation both in presence and absence of baicalein by using a WAKO LabAssay Triglyceride Kit (Wako Pure Chemical). Protein concentration was determined by using a Pierce BCA Protein Assay Kit (Thermo Scientific, Rockford, IL, USA). Intracellular triglyceride level was adjusted with the protein concentration.

### Quantitative PCR (qPCR)

RNA was extracted as described previously [14], and processed for cDNA synthesis by the use of random primers (Takara-Bio, Kyoto, Japan) and ReverTra Ace Reverse Transcriptase (TOYOBO, Osaka, Japan). Quantitative PCR (qPCR) analysis was performed on a LightCycler System (Roche Diagnostics, Mannheim, Germany) with THUNDERBIRD SYBR PCR Mater Mix (TOYOBO) or StepOne Plus Real-Time PCR System (Thermo Scientific) with Power SYBR Green Master Mix (Thermo Scientific). The nucleotide sequences of the gene-specific primers were listed in S1 Table. The mRNA level of the genes was normalized to that of the TATA-binding protein (TBP).

### Measurement of glycerol level

3T3-L1 cells were differentiated into adipose cells as described above for 5 days in DMEM containing baicalein or not. At 5 days after the initiation of adipogenesis, the medium was changed to phenol red-free DMEM (Sigma) containing insulin with or without baicalein. At day 6, the glycerol content released to the medium was measured by using Free Glycerol Assay Reagent (Cayman Chemical).

### Western blot analysis

Cell extracts (crude proteins) were prepared as described previously [13], and separated by SDS-PAGE gels, followed by transferred onto Immobilon PVDF membranes (Millipore, Bedford, MA, USA). The membranes were incubated with primary antibodies. After washing the membranes, they were incubated with the horseradish peroxidase-conjugated secondary antibody. Immunoreactive proteins were detected using a Pierce Western Blotting Substrate (Thermo Scientific) and a Luminoimaging Analyzer LAS-3000, and the signals were analyzed by Multi Gauge software (Fujifilm, Tokyo, Japan).

### Chromatin immunoprecipitation (ChIP) assay

We employed a ChIP assay to analyze the binding of C/EBPα protein to the *cis*-element of the GLUT4 gene promoter [15] by using anti-C/EBPα antibody or normal goat IgG. After immunoprecipitation and purification, DNAs were used for PCR analysis with the gene-specific primers: 5′-GCAGGCGGGAACCTTAGGGGCG-3′ and 5′-CCAAGGCTCTCCGGGATCTAGTG-3′. PCR was carried out by the use of KOD FX DNA polymerase (TOYOBO), and conducted by the following conditions: initial denaturation at 94°C for 2 min, followed by 35 cycles of 98°C for 10 sec, 55°C for 30 sec, and 68°C for 20 sec. PCR products were analyzed by agarose gel electrophoresis.

### 2-NBDG uptake assay

3T3-L1 cells were differentiated into adipose cells for 6 days in medium with or without baicalein. At day 6, the cells were washed with PBS(-), followed by incubated in PBS(-) with a fluorescent glucose analog 2-NBDG (150 μg/ml) for 30 min at 37°C in CO_2_ incubator. Thereafter the cells were washed again with PBS(-) and resuspended in it, and their fluorescence was observed with a fluorescence microscope (CKX-41FL, Olympus, Tokyo, Japan).

The cells were seeded onto black 96-well plates for 24 h, and differentiated into adipocytes and treated with 2-NBDG as described above. The fluorescence was measured by the use of a multimode Enspire 2300 plate reader (excitation = 485 nm, emission = 535 nm, PerkinElmer, Waltham, MA, USA). After that, the cells were suspended with PBS(-) and sonicated to disrupt them, followed by centrifugation to remove cell debris. The resultant supernatant (crude cell extracts) was used to determine protein concentration. The 2-NBDG uptake level was calculated by normalization of the protein concentration.

### Expression of mouse GLUT4

To construct the expression vector for mouse GLUT4, the open reading frame of mouse GLUT4 was amplified by PCR with KOD-Plus- DNA Polymerase (TOYOBO). The resultant PCR products were cloned into the pcDNA3.1 vector (Thermo Fischer Scientific). The inserted GLUT4 fragment was verified by nucleotide sequencing.

3T3-L1 cells were transfected with the GLUT4 expression plasmid, pCMV-mGLUT4 by the use of the Neon Transfection system (Thermo Fischer Scientific). After 24 h, cells were differentiated into adipocytes as described above.

### Statistical analysis

The results are expressed as mean ± S.D. Comparison of 2 treatment groups was analyzed by performing Student’s *t*-test. To evaluate variances among multiple groups, one-way ANOVA followed by Tukey’s post-hoc test were performed. *p*<0.05 was considered statistically significant.

## Results

### Cell-toxic effect of baicalein toward 3T3-L1 adipocytes

We cultured 3T3-L1 cells for 6 days in medium containing various concentrations of baicalein (0–100 μM), and measured the cell toxicity of baicalein. No significant effect of it on cell viability was observed up to 50 μM baicalein (Fig 1B). In contrast, the cell viability lowered by about 24% of the vehicle-treated 3T3-L1 cells, when the cells were incubated in medium with 100 μM baicalein (Fig 1B).

Then, we studied the effects of baicalein on adipocyte differentiation. 3T3-L1 cells were differentiated into adipose cells for 6 days in medium without or with baicalein (0 or 50 μM), and the accumulated lipids in the cells were visualized by staining with Oil Red O. The intracellular lipid droplets elevated during adipocyte differentiation in the absence of baicalein (Fig 1C). In contrast, they were lowered when the cells were differentiated into adipocytes in medium in the presence of baicalein (50 μM; Fig 1C). Moreover, the triglyceride content in the differentiated cells was elevated about 8.4-fold, as compared with the undifferentiated 3T3-L1 cells (Fig 1D). However, when the cells were differentiated into adipose cells for 6 days in medium with baicalein (50 μM), the triglyceride level in the cells was lowered to about 18% of the vehicle-treated differentiated cells (Fig 1D). In addition, 10 μM baicalein did not suppress the lipid accumulation in adipocytes (data not shown). These results suggest that baicalein lowered the intracellular lipid accumulation in 3T3-L1 adipocytes.

### Decreased expression level of adipogenic and lipogenic genes by baicalein

**Fig 2:**
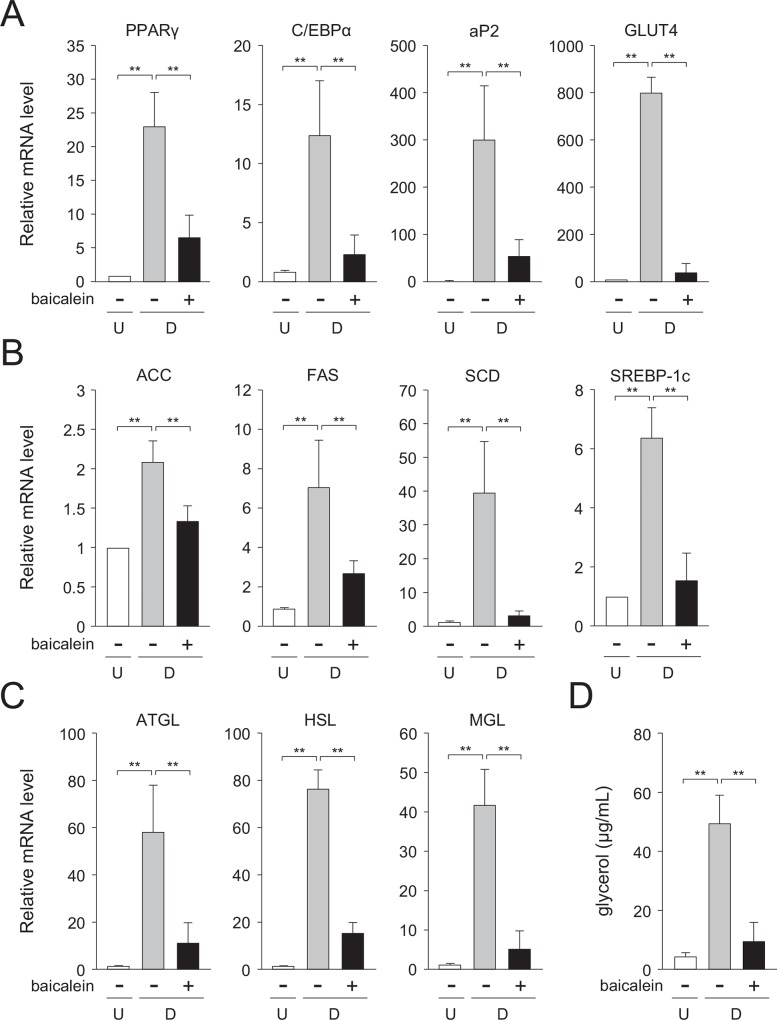
Suppressed expression of adipogenesis-related genes in baicalein-treated 3T3-L1 cells. A, Transcription level of the adipogenic genes in baicalein-treated cells. 3T3-L1 cells (undifferentiated cells: U; *white columns*) were differentiated into adipocytes (differentiated cells: D) for 6 days in DMEM in the absence (*gray columns*) or presence of baicalein (50 μM; *black columns*). Data are the means ± S.D. from three experiments. **p<0.01, as indicated by the brackets. B, Expression level of the lipogenic genes in baicalein-treated 3T3-L1 cells. Cells were differentiated into adipocytes as described in the legend of Fig 2A. Data are the means ± S.D. from three experiments. **p<0.01, as indicated by the brackets. C, Messenger RNA level of the lipolytic genes in baicalein-treated cells. 3T3-L1 cells were differentiated into adipocytes as described in the legend of Fig 2A. Data are the means ± S.D. from three experiments. **p<0.01, as indicated by the brackets. D, Change in glycerol level in baicalein-treated 3T3-L1 cells. Cells were differentiated into adipocytes as described in Materials and Methods. Data are the means ± S.D. from three experiments. **p<0.01, as indicated by the brackets.

The transcription levels of the adipogenic genes; i.e., the PPARγ, C/EBPα, fatty acid binding protein 4 (aP2), and GLUT4 genes were elevated approximately 23-, 12-, 299-, and 799-fold, respectively, as compared with the undifferentiated 3T3-L1 cells (Fig 2A). Whereas, when the cells were differentiated into adipose cells for 6 days in medium with baicalein, each of their mRNA levels was decreased to about 28, 18, 18, and 5.1%, respectively, of the vehicle-treated differentiated cells (Fig 2A).

We also examined the change in the transcription levels of the acetyl-CoA carboxylase (ACC), fatty acid synthase (FAS), stearoyl-CoA desaturase (SCD), and SREBP-1c genes involved in the lipogenesis. 3T3-L1 cells were differentiated into adipose cells for 6 days in medium containing baicalein or not. The mRNA levels of the ACC, FAS, SCD, and SREBP-1c genes were elevated approximately 2.1-, 7.1-, 40-, and 6.4-fold, respectively, during adipocyte differentiation (Fig 2B). Whereas, when the cells were differentiated into adipose cells for 6 days in medium in the presence of baicalein, each of their expression levels was reduced by approximately 36, 60, 92, and 76%, respectively, of the vehicle-treated differentiated cells (Fig 2B). These results reveal that the expression levels of both adipogenic and lipogenic genes were lowered by the treatment with baicalein in 3T3-L1 adipocytes.

### Reduction in lipolysis by baicalein

Triglyceride is converted to glycerol and fatty acids through the sequential reactions of adipocyte triglyceride lipase (ATGL), hormone-sensitive lipase (HSL), and monoacylglyceride lipase (MGL) in the lipolytic process. We measured the expression levels of these lipolytic genes in baicalein-treated adipose cells, and also measured the level of glycerol released from adipocytes. The transcription levels of the lipolytic ATGL, HSL, and MGL genes were elevated approximately 58-, 76-, and 42-fold, respectively, when we differentiated the cells into adipocytes (Fig 2C). On the contrary, when the cells were differentiated into adipose cells in medium containing baicalein for 6 days, their expression levels were lowered to approximately 18, 23, and 11%, respectively, of the vehicle-treated differentiated cells (Fig 2C). Furthermore, the level of glycerol released from the differentiated cells was increased approximately 8.2-fold, as compared with the undifferentiated 3T3-L1 cells (Fig 2D). Whereas, when baicalein was added into the medium, the released glycerol content was reduced to approximately 20% of the vehicle-treated differentiated cells (Fig 2D). These results mean that baicalein lowered lipolysis in 3T3-L1 adipocytes.

**Fig 3:**
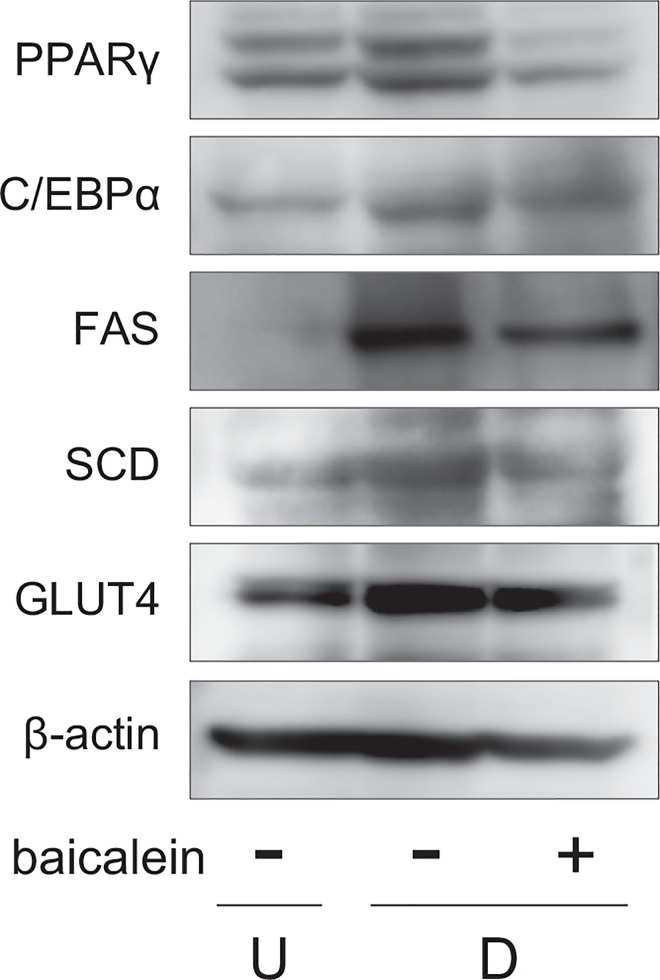
Change of expression of proteins associated with adipogenesis in baicalein-treated 3T3-L1 cells. 3T3-L1 cells were differentiated as described in Fig 2A. Protein levels were detected by Western blot analysis using cell extracts (15 μg/lane).

Moreover, we investigated the protein expression in Western blot analysis. The expression of PPARγ, C/EBPα, FAS, SCD, and GLUT4 was enhanced during adipogenesis, and their levels were lowered by the treatment with baicalein (Fig 3).

### Differentiation stage-specific suppression of adipogenesis by baicalein

**Fig 4:**
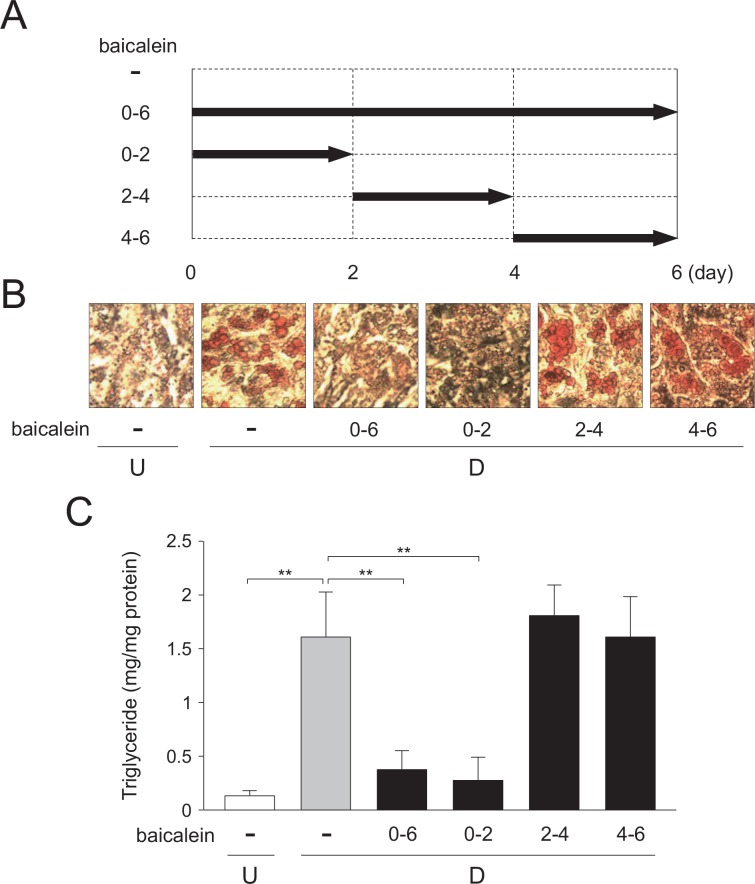
Differentiation stage-specific effects of baicalein during adipogenesis. A, Addition of baicalein at the indicated time-points during adipocyte differentiation of 3T3-L1 cells for 6 days. B, Oil Red O staining of intracellular lipids in 3T3-L1 cells in DMEM with stage-specific addition of baicalein. The cells (undifferentiated cells: U; *white column*) were differentiated into adipocytes (differentiated cells: D) for 6 days in DMEM without (*gray column*) or with (50 μM; *black columns*) baicalein during days 0–6, 0–2, 2–4, or 4–6. C, Intracellular triglyceride level. 3T3-L1 cells (undifferentiated cells: U) were differentiated into adipocytes (differentiated cells: D) as described in the legend of Fig 3B. Data are the means ± S.D. from three experiments. **p<0.01, as indicated by the brackets.

Next, we elucidated the molecular mechanism of baicalein-mediated inhibition of adipogenesis in 3T3-L1 adipocytes. At first, we investigated the effect of baicalein on adipogenesis at different stages of the differentiation process. Adipogenesis (for 6 days; days 0–6) was divided into 3 stages: early stage (day 0–2) at clonal expansion stage; middle stage (day 2–4) at initiation of adipogenesis, and late stage (after day 4) at maturation of adipocytes. 3T3-L1 cells were differentiated into adipose cells in medium containing baicalein at the indicated stages (Fig 4A). On day 6, the intracellular lipid-droplets were visualized by staining with Oil Red O, and the triglyceride levels in the cells were also measured. When baicalein was present during days 0–2 or days 0–6 of 6-day-adipogenesis, the accumulation of lipids in the cells was decreased as compared with the vehicle-treated differentiated cells (Fig 4B and 4C). In contrast, when baicalein was present in the medium during days 2–4 or days 4–6 in 6-day-adipogenesis, the intracellular lipid levels were almost the same as that for the vehicle-treated differentiated cells (Fig 4B and 4C). Therefore, these results reveal that baicalein repressed the intracellular lipid accumulation during the early stage of adipogenesis.

**Fig 5:**
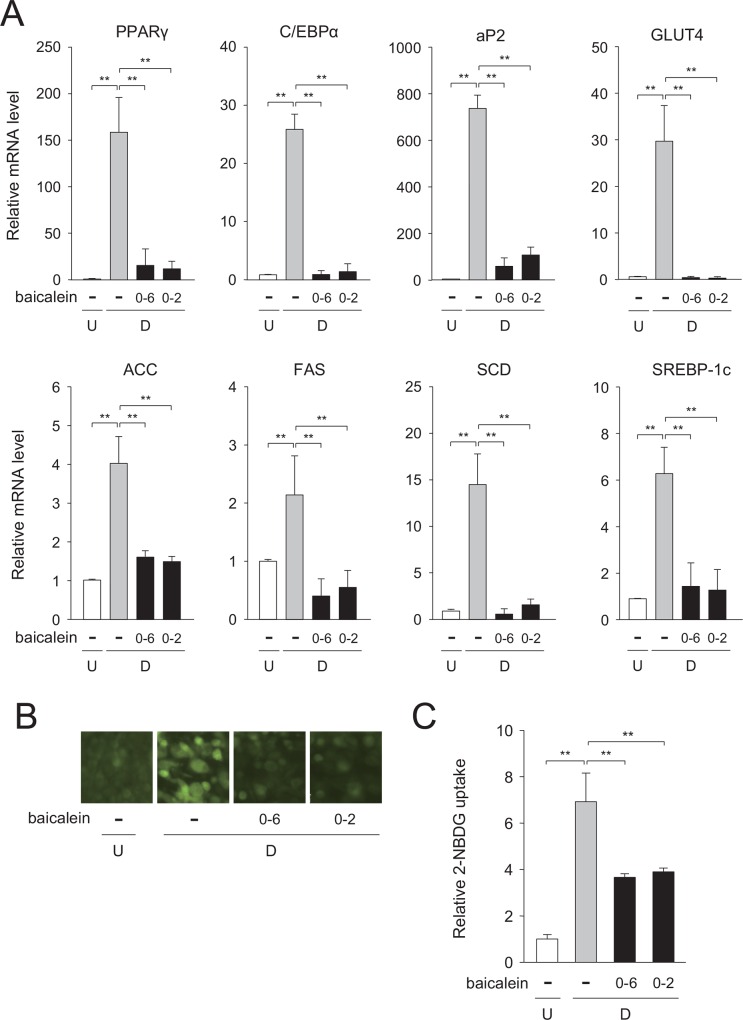
Decreased expression of adipogenesis-related genes by baicalein in early stage of adipogenesis. A, Expression levels of the adipogenic and lipogenic genes. 3T3-L1 cells (undifferentiated cells: U; *white columns*) were differentiated into adipocytes (differentiated cells: D) for 6 days in DMEM in the absence (*gray columns*) or presence (50 μM; *black columns*) of baicalein during days 0–2 or 0–6. Data are presented as the means ± S.D. from three experiments. **p<0.01, as indicated by the brackets. B, Decrease in glucose uptake in 3T3-L1 cells by baicalein. Cells (undifferentiated cells: U) were differentiated into adipocytes (differentiated cells: D) for 6 days in DMEM containing baicalein or not (0 or 50 μM) during days 0–2 or 0–6. Cells were further incubated with the fluorescent 2-NBDG, and then observed under a fluorescence microscope. C, Quantification of glucose uptake in baicalein-treated, differentiated 3T3-L1 cells. Cells were prepared as described in the legend of Fig 5B. The data are presented as the value relative to that of the undifferentiated cells and are shown as the means ± S.D. from three experiments. **p<0.01, as indicated by the brackets.

Next, we measured changes in the expression levels of the adipogenic and lipogenic genes in cells which were differentiating into adipocytes in medium containing baicalein or not during days 0–2, days 0–6 during the 6-day-adipogenesis. The transcription levels of the adipogenic PPARγ, C/EBPα, and aP2 genes and of the lipogenic ACC, FAS, and SCD genes were all decreased when the cells were treated with baicalein during days 0–2 of adipogenesis, which results were almost the same as those obtained when baicalein was present for all 6 days (Fig 5A) and the results obtained in medium containing baicalein during days 2–4 or days 4–6 during the 6-day-adipogenesis (data not shown).

Glucose uptake is critical in the regulation of the glucose metabolism in the cells. Glucose is incorporated into the cells through GLUT4 in adipocytes. So next, we investigated the uptake of 2-NBDG, a fluorescent glucose analogue, into the cells. 3T3-L1 cells were differentiated into adipose cells for 6 days in medium with baicalein present during days 0–2, days 0–6, or absent during the adipogenesis. The level of 2-NBDG uptake was increased during adipogenesis (Fig 5B and 5C), and this elevation was suppressed when the cells were treated with baicalein during days 0–2 or days 0–6 during the adipogenesis process (Fig 5B and 5C). These results mean that baicalein inhibited the early stage of adipogenesis via suppression of GLUT4-mediated glucose uptake in adipocytes.

### Baicalein did not affect the C/EBPβ and C/EBPδ gene expression in the early stage of adipogenesis

C/EBPβ and C/EBPδ are known as the transcription factors that play central roles in the regulation of the early stage of adipogenesis [4, 16]. They sequentially activate the expression of the PPARγ and C/EBPα genes, both of which are crucial transcription factors in adipocytes [4, 16]. To elucidate the molecular mechanism of baicalein-suppressed the lipid accumulation in 3T3-L1 adipocytes, we studied the effects of baicalein on the transcription of the C/EBPβ and C/EBPδ genes in the early stage of adipogenesis. The mRNA levels of both of them were upregulated within 1 h after the initiation of incubation in DMEM containing MDI, and these levels gradually decreased during adipogenesis for 24 h (S1A Fig). However, no significant variances were detected between baicalein-treated and non-treated cells in each of their expression levels during adipogenesis for 24 h (S1A Fig). Almost the same results were obtained in Western blot analysis (S1B Fig). These results reveal that the expression of the C/EBPβ and C/EBPδ genes in the early stage of adipogenesis was not affected by the treatment with baicalein in adipocytes.

### Suppression of glucose uptake through decreased C/EBPα-activated GLUT4 expression by baicalein

**Fig 6:**
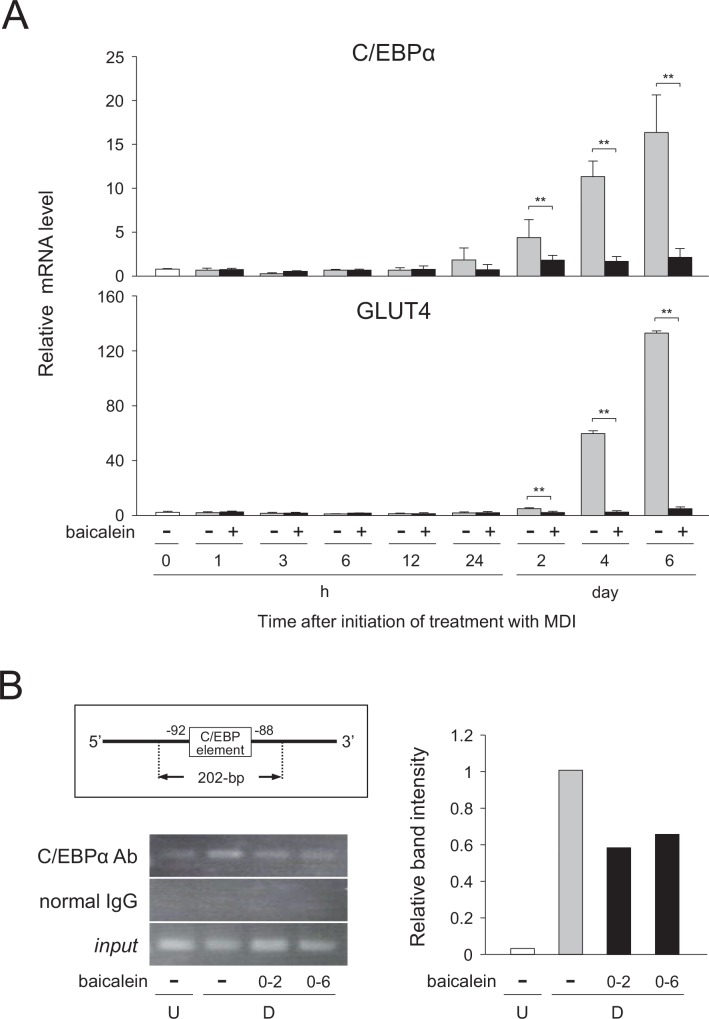
Regulation of C/EBPα-mediated expression of GLUT4 gene in baicalein-treated 3T3-L1 cells. A, 3T3-L1 cells (undifferentiated cells: U; *white columns*) were differentiated into adipocytes (differentiated cells: D) for 6 days in DMEM without (*gray columns*) or with (50 μM; *black columns*) baicalein. The mRNA levels were quantified by qPCR. Data are presented as the means ± S.D. from three experiments. **p<0.01, as indicated by the brackets. B, ChIP assay. 3T3-L1 cells (undifferentiated cells: U) were differentiated into adipocytes (differentiated cells: D) for 6 days in DMEM without or with baicalein (0 or 50 μM) during days 0–2 or 0–6, and the ChIP assay was then performed. The resultant PCR products were analyzed by agarose-gel electrophoresis. *input* (input control) means that a diluted aliquot before immunoprecipitation was used for PCR amplification. The results are representative from three experiments. Band intensity was measured with MultiGauge software.

To further analyze the molecular mechanism of the lipid accumulation decreased by baicalein in adipocytes, we investigated the glucose uptake and the mechanism of the transcriptional regulation of the GLUT4 gene in the baicalein-treated differentiated cells. Baicalein lowered the transcription levels of the C/EBPα and GLUT4 genes (Figs 2A, 3 and 5A). Moreover, it has been reported that the expression of the GLUT4 gene is regulated by C/EBPα in adipocytes [13, 15, 17]. Thus, we investigated whether the C/EBPα-GLUT4 signaling is involved in the control of the baicalein-decreased intracellular lipid accumulation. At first, we studied the time-course change in the mRNA levels of the C/EBPα and GLUT4 genes during adipogenesis. When 3T3-L1 cells were differentiated into adipose cells for 6 days in medium with or without baicalein, the mRNA level of the C/EBPα gene was gradually increased after 24 h of the initiation of adipogenesis (Fig 6A). However, when the cells were differentiated into adipose cells for 6 days in medium containing baicalein, its mRNA level was clearly suppressed (Fig 6A). Furthermore, almost the same expression profiles were found for the expression of the GLUT4 gene during adipogenesis in either the presence or absence of baicalein (Fig 6A).

Next, the binding of C/EBPα protein to the *cis*-element of the GLUT4 gene promoter was investigated by a ChIP assay. The expected size (202-bp; Fig 6B) of an amplicon harboring the C/EBP-binding element at -92 of the GLUT4 promoter region was detected in the formaldehyde-linked DNA-protein complexes immunoprecipitated by using anti-C/EBPα antibody (Fig 6B). The ability of C/EBPα protein to bind the C/EBP-binding element of the GLUT4 promoter region was enhanced during adipogenesis (Fig 6B); whereas, when the cells were differentiated into adipose cells in medium with baicalein, its binding level was reduced (Fig 6B). In contrast, when normal goat IgG was added in place of anti-C/EBPα antibody, no detectable signal was observed, although signals of the expected size were detected in all *input* samples (Fig 6B). These results reveal that the expression of the C/EBPα and GLUT4 genes was suppressed by baicalein in a similar manner during adipogenesis. Moreover, baicalein decreased the binding level of C/EBPα protein to the corresponding *cis*-element of the GLUT4 gene promoter, thus indicating that baicalein inhibited the C/EBPα-GLUT4 pathway in adipocytes.

### Inhibition of Akt activation by baicalein in the early stage of adipogenesis

**Fig 7:**
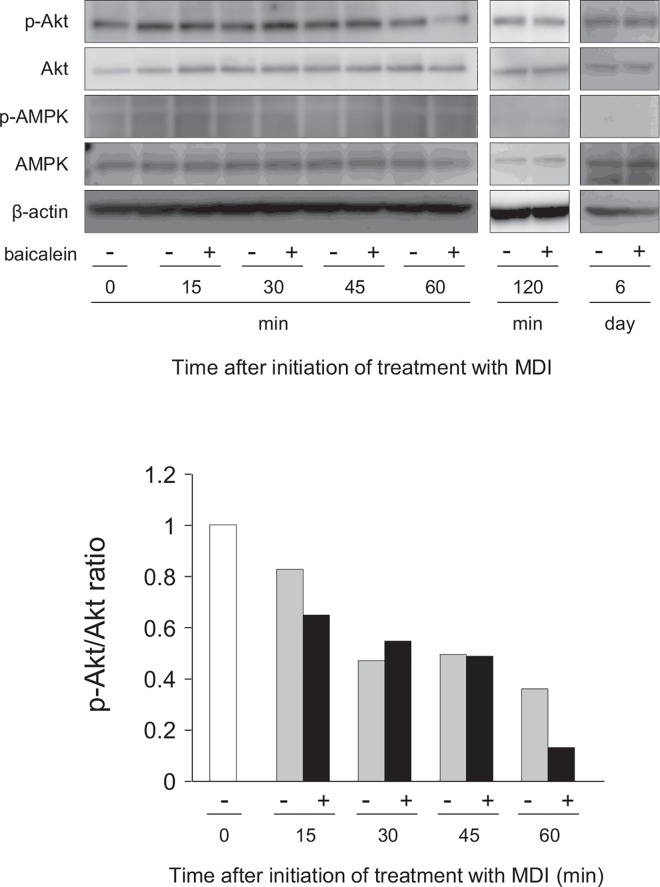
Inhibition of Akt phosphorylation in 3T3-L1 cells by baicalein. A, 3T3-L1 cells were differentiated into adipocytes for 60 min, 120 min or 6 days in DMEM containing MDI with baicalein or not (0 or 50 μM). Cell lysates (15 μg/lane) were subjected to SDS-PAGE and Western blot analysis for the detection of phosphorylated Akt (p-Akt), Akt, phosphorylated AMPK (p-AMPK) and AMPK proteins. β-actin was also detected as the internal control. The results are representative from at least three experiments. B, Ratio of band intensities of p-Akt/Akt. Band intensity was measured with MultiGauge software. The ratio of p-Akt and total Akt was shown.

Some of natural compounds such as flavonoids are involved in the regulation of the early stage of adipogenesis [18], and control the insulin signaling in adipocytes [19, 20]. In addition, activation of Akt and AMPK is regulated by the insulin signaling [21]. So we examined whether the insulin signaling pathway was involved in the baicalein-inhibited adipogenesis or not. 3T3-L1 cells were differentiated into adipose cells for 6 days in DMEM containing baicalein, after which the expression levels of Akt and AMPK, and of their phosphorylated proteins were examined by Western blot analysis. Akt was expressed in the undifferentiated cells (0 h), and its expression level was slightly increased during adipocyte differentiation (Fig 7A and 7B). Akt was continuously phosphorylated during adipogenesis for 60 min. However, its phosphorylation was suppressed by baicalein at 60 min (1 h) after the starting of adipogenesis. In contrast, AMPK was slightly phosphorylated, but not altered in its phosphorylation level during adipogenesis for 60 min; although the expression of AMPK was detected in all samples (Fig 7A and 7B). In contrast, at 120 min and 6 days after the starting of adipogenesis, the transcription levels of Akt and AMPK, and of their phosphorylated forms were the same in both baicalein-treated and untreated differentiated cells (Fig 7A and 7B). These results mean that baicalein lowered the phosphorylation of Akt in the very early stage of adipogenesis of 3T3-L1 cells (at 1 h after the initiation of adipogenesis).

### Inhibition of Akt activation in the very early stage of adipogenesis reduces intracellular lipids through decreased glucose uptake

**Fig 8:**
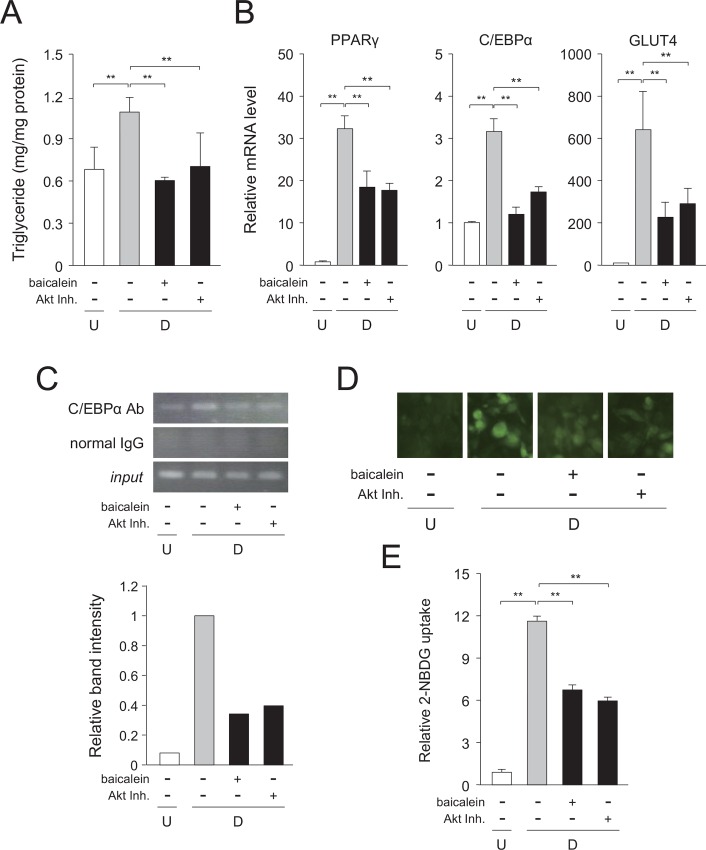
Inhibition of very early stage of adipogenesis in 3T3-L1 cells by baicalein or Akt inhibitor. A, Intracellular triglyceride level. 3T3-L1 cells (undifferentiated cells: U; *white column*) were differentiated into adipocytes (differentiated cells: D; *gray column*) for 6 days and treated with baicalein or an Akt inhibitor (Akt Inh.; *black columns*) for the initial 1.5 h of adipogenesis. Data are presented as the means ± S.D. from three experiments. **p<0.01, as indicated by the brackets. B, Expression levels of adipogenic genes. Cells were cultured as described in the legend of Fig 8A. Data are shown as the means ± S.D. from three experiments. **p<0.01, as indicated by the brackets. C, ChIP assay. 3T3-L1 cells were cultured as described in the legend of Fig 8A. The results are representative from three experiments. Band intensity was measured with MultiGauge software. D, Change in glucose uptake. 3T3-L1 cells were cultured as described in the legend of Fig 8A. Cells were then incubated with fluorescent 2-NBDG, and then observed under a fluorescence microscope. The results are representative from three experiments. E, Quantification of glucose uptake in baicalein- or Akt inhibitor (Akt Inh.)-treated 3T3-L1 cells. The data are presented as the value relative to that of the undifferentiated cells and are shown as the means ± S.D. from three experiments **p<0.01, as indicated by the brackets.

To further confirm the importance of the suppression of Akt activation in the very early stage of adipogenesis, we treated the cells with baicalein or an Akt inhibitor (Akt Inh.) for 1.5 h after the initiation of adipogenesis during 6-day-adipocyte differentiation process. To completely inhibit Akt activation at around 1 h after the initiation of adipogenesis, we treated the cells with baicalein or an Akt inhibitor for 1.5 h. 3T3-L1 cells were differentiated into adipose cells in medium with MDI and baicalein or Akt inhibitor for 1.5 h after the initiation of adipogenesis, and the cells were continued to differentiate into adipose cells for 6 days in medium with MDI or insulin alone. The triglyceride level in the cells was decreased by the treatment with baicalein or Akt inhibitor for 1.5 h after the initiation of adipogenesis during 6-day-adipocyte differentiation process (Fig 8A). In addition, the expression levels of the PPARγ, C/EBPα, and GLUT4 genes were reduced by either treatment (Fig 8B). Moreover, these treatments reduced the binding of C/EBPα to the GLUT4 gene promoter after 6 days of adipocyte differentiation (Fig 8C). In addition, the treatment of Akt inhibitor did not affect the expression of C/EBPβ and C/EBPδ genes (data not shown). Furthermore, the glucose uptake into the cells were also repressed by either treatment (Fig 8D and 8E).

### Rescue study of baicalein-lowered glucose uptake by expression of GLUT4

**Fig 9:**
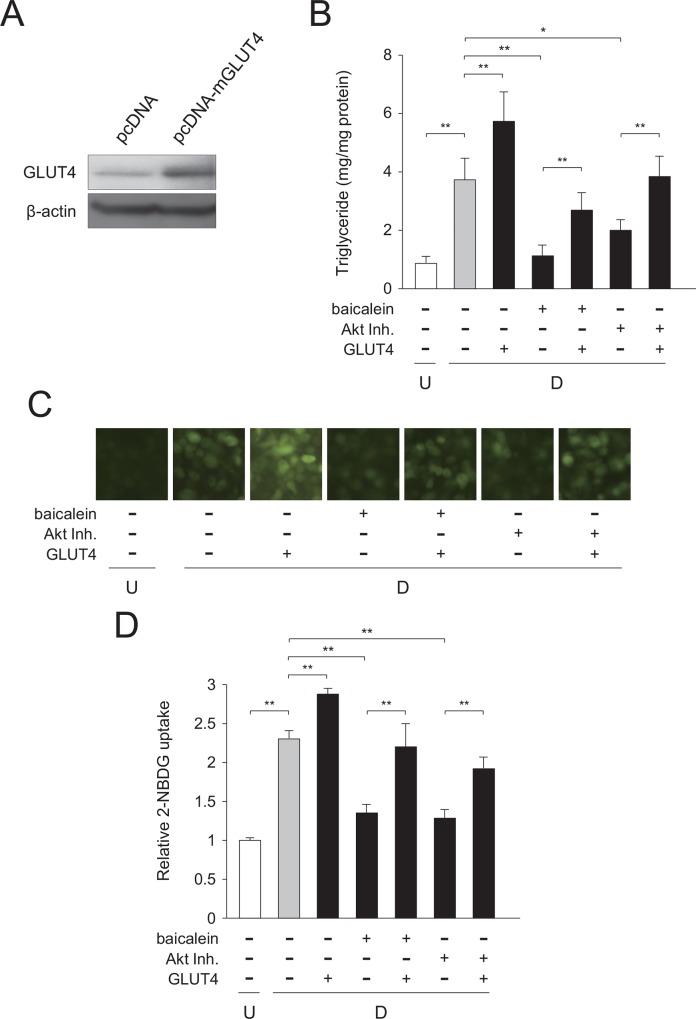
Expression of GLUT4 in baicalein- or Akt inhibitor-treated 3T3-L1 cells. A, Expression of the recombinant mouse GLUT4 protein in 3T3-L1 cells. 3T3-L1 cells were transfected with GLUT4 (pcDNA-mGLUT4) or the empty (pcDNA) vector. GLUT4 levels were detected by Western blot analysis using cell extracts (15 μg/lane). B, Intracellular triglyceride level in GLUT4-transfected cells. 3T3-L1 cells were transfected with GLUT4 vector by electroporation. After 24 h, cells (undifferentiated cells: U; *white column*) were differentiated into adipocytes (differentiated cells: D; *gray column*) for 6 days and treated with baicalein or an Akt inhibitor (Akt Inh.; *black columns*) for the initial 1.5 h of adipogenesis. Data are presented as the means ± S.D. from three experiments. *p<0.05, **p<0.01, as indicated by the brackets. C, Change in glucose uptake in GLUT4-transfected cells. 3T3-L1 cells were cultured as described in the legend of Fig 9B. Cells were then incubated with fluorescent 2-NBDG, and then observed under a fluorescence microscope. The results are representative from three experiments. D, Measurement of glucose uptake in GLUT4-transfected cells. Data are presented as the value relative to that of the undifferentiated cells and are shown as the means ± S.D. from three experiments **p<0.01, as indicated by the brackets.

To obtain the further evidence that baicalein or Akt inhibitor suppress the very early stage of adipogenesis by decreased intracellular triglyceride level through reduced Akt-C/EBPα-GLUT4-mediated glucose uptake in adipocytes, we performed the rescue study by expressing GLUT4 (Fig 9A). 3T3-L1 cells were transfected with the GLUT4 expression vector. After that, the cells were caused to differentiate into adipocytes in medium with MDI and baicalein or Akt inhibitor for 1.5 h after the initiation of adipogenesis. Baicalein- or Akt inhibitor-mediated reduction of intracellular triglyceride levels were mostly cleared by the expression of GLUT4 (Fig 9B). Moreover, the suppression of glucose uptake into the cells by either treatment was negated by expressing GLUT4 (Fig 9C and 9D). These results, taken together, indicate that suppression of the very early stage of adipogenesis by baicalein or Akt inhibitor occurred by decreased intracellular triglyceride levels through reduced Akt-C/EBPα-GLUT4-mediated glucose uptake in adipocytes.

## Discussion

Obesity (adipogenesis) is understood as abnormal or excessive fat accumulation, and is one of trigger factors for the development of various diseases such as hypertension, type 2 diabetes, cancer, and coronary heart disease [2]. Thus, elucidation of the mechanisms regulating obesity (adipogenesis) is important to control the progression of adipogenesis.

Japanese and Chinese traditional medicines have various physiological effects and not a few of them have been used clinically [22, 23]. They include a number of constituents such as flavonoids, alkaloids, polysaccharides, terpenes, and sterols. Plant flavonoids such as genistein, avicularin, and apigenin decrease the intracellular lipid accumulation in adipocytes [6, 13, 15]. Thus, several plant-derived substances have the potential for repression of adipogenesis. In the present study, we found that a plant-derived baicalein significantly inhibited the intracellular lipid accumulation in mouse 3T3-L1 adipocytes. Our current data suggest that baicalein-mediated suppression of this intracellular lipid accumulation occurred via inhibition of C/EBPα-GLUT4-activated glucose uptake through the Akt signaling in the very early stage of adipogenesis.

Baicalein is a cell membrane-permeable flavone, a type of flavonoid, and the aglycone of baicalin, which has been isolated from the roots of *S*. *baicalensis*. Baicalein is one ingredient of the Japanese herbal medicine, Sho-saiko-to that has been administered to patients with liver diseases [24]. Moreover, baicalein represses the intracellular lipid accumulation by negatively regulating the early stage of adipogenesis (within 2 days after the initiation of adipogenesis) [25, 26]. Consistent with these reports, our present results showed that baicalein suppressed the intracellular lipid accumulation in the very early stage of adipogenesis (within 1 h after the starting adipogenesis) through repression of the Akt-C/EBPα-GLUT4 pathway (Figs 8 and 9).

Insulin induces the accumulation of intracellular lipids with the sequential activation of adipogenesis-related transcription factors [27]. Moreover, insulin regulates the activity of the phosphatidylinositol 3-kinase (PI3K)/Akt-mediated signaling [21, 28, 29], causing the accumulation of intracellular lipids [30–32]. Akt is an important factor in the regulation of adipocyte differentiation, and its phosphorylation is activated by insulin [29, 33], which phosphorylated form enhances adipogenesis [28]. Moreover, RNAi-mediated knockdown of Akt mRNA expression suppresses adipogenesis with lowered expression of PPARγ and C/EBPα [34, 35]. In addition, it has been reported that baicalein inhibits lipid accumulation through mammalian target of rapamycin (mTOR) signaling via cell cycle arrest in the G_0_/G_1_ stage, which is downstream signaling of Akt [25]. Therefore, mTOR-suppressed accumulation of intracellular lipids by baicalein occurs via the suppression of Akt activation in 3T3-L1 adipocytes.

Glucose uptake into cells is regulated by GLUT proteins in mammals [36]. The GLUT4 is responsible for glucose incorporation into muscle and adipocytes, and GLUT4-mediated glucose uptake is an important step in the control of glucose metabolism and is stimulated by insulin [37]. GLUT4 siRNA decreased the lipid drop-let in 3T3-L1 cells. GLUT4 is required for lipogenesis in the differentiated adipocytes [38]. Baicalein lowered the mRNA level of the GLUT4 gene in adipocytes through the inhibition of Akt activation in the very early stage of adipogenesis (Fig 8). Moreover, the ChIP assay showed that C/EBPα bound to the GLUT4 gene promoter (Fig 6B). Baicalein repressed the transcription of the GLUT4 and C/EBPα genes (Figs 5A, 6A and 8B) and reduced glucose uptake (Figs 5B, 5C, 8D and 8E). In addition, GLUT4 expression is enhanced by C/EBP proteins [17], indicating that baicalein inhibited C/EBPα-mediated GLUT4 expression and subsequent glucose uptake into the cells. Furthermore, expression of GLUT4 protein was clearly rescued the baicalein-reduced GLUT4-mediated glucose uptake in 3T3-L1 cells (Fig 9). Therefore, baicalein inhibited the intracellular lipid accumulation in the very early stage of adipogenesis by repressing the Akt-C/EBPα-GLUT4-activated glucose uptake in 3T3-L1 adipocytes. However, only initial short-time treatment with baicalein showed the drastic reduction of lipid accumulation. Phosphorylation of Akt was suppressed at 60 min, but not at 120 min by the treatment with baicalein (Fig 7). Thus, another suppression mechanism might be involved in the baicalein-mediated reduction of intracellular lipid accumulation. Further study is needed to solve this concern.

In conclusion, we demonstrated that a plant flavonoid baicalein repressed the intracellular lipid accumulation by lowering Akt-C/EBPα-GLUT4-activated glucose uptake in the very early stage of adipogenesis of 3T3-L1 cells. Thus, the Akt signaling is critical in the progression of adipogenesis in the very early stage of adipogenesis. In a future study, we have to elucidate the *in vivo* function of baicalein as an anti-obesity agent. In addition, more precise repression mechanisms by baicalein-inhibited Akt activation should be investigated.

## Supporting Information

S1 Fig Change in expression of C/EBPβ and δ genes by baicalein in the early stage of adipogenesis. A. 3T3-L1 cells (undifferentiated cells: U; *white columns*) were differentiated into adipocytes (differentiated cells: D) for 24 h in DMEM without (*gray columns*) or with (50 μM; *black columns*) baicalein. The mRNA levels were quantified by qPCR. Data are presented as the means ± S.D. **p<0.01, as indicated by the brackets. B. 3T3-L1 cells were differentiated as described in S1A Fig. Protein levels were detected by Western blot analysis using cell extracts (15 μg/lane).
(DOC)

S1 Table Nucleotide sequences of primers used in qPCR (DOC)
