# Supplementary material for: Suppression of Very Early Stage Of Adipogenesis by Baicalein, a Plant-Derived Flavonoid through Reduced Akt-C/EBPα-GLUT4 Signaling-Mediated Glucose Uptake in 3T3-L1 Adipocytes
Source: PLoS One. 2016 Sep 26;11(9):e0163640. doi: 10.1371/journal.pone.0163640 (PMC5036867; doi:10.1371/journal.pone.0163640)
Supplement: S1 Fig — A. 3T3-L1 cells (undifferentiated cells: U; white columns) were differentiated into adipocytes (differentiated cells: D) for 24 h in DMEM without (gray columns) or with (50 μM; black columns) baicalein. The mRNA levels were quantified by qPCR. Data are presented as the means ± S.D. **p<0.01, as indicated by the brackets. B. 3T3-L1 cells were differentiated as described in S1A Fig. Protein levels were detected by Western blot analysis using cell extracts (15 μg/lane). (DOC) [file pone.0163640.s001.doc]

**S1 Fig. Change in expression of C/EBPβ and δ genes by baicalein in the early stage of adipogenesis**

A. 3T3-L1 cells (undifferentiated cells: U; *white columns*) were differentiated into adipocytes (differentiated cells: D) for 24 h in DMEM without (*gray columns*) or with (50 μM; *black columns*) baicalein. The mRNA levels were quantified by qPCR. Data are presented as the means ± S.D. ***p*<0.01, as indicated by the brackets. B. 3T3-L1 cells were differentiated as described in S1A Fig~~. S1A~~. Protein levels were detected by Western blot analysis using cell extracts (15 μg/lane).
