# Supplementary material for: Suppression of Very Early Stage Of Adipogenesis by Baicalein, a Plant-Derived Flavonoid through Reduced Akt-C/EBPα-GLUT4 Signaling-Mediated Glucose Uptake in 3T3-L1 Adipocytes
Source: PLoS One. 2016 Sep 26;11(9):e0163640. doi: 10.1371/journal.pone.0163640 (PMC5036867; doi:10.1371/journal.pone.0163640)
Supplement: S1 Table — (DOC) [file pone.0163640.s002.doc]

**S1 Table. Nucleotide sequences of primers used in qPCR**

Gene Acc.No. 　Forward 　　 Reverse

PPARγ NM_011146 5’-CAAGAATACCAAAGTGCGATCAA-3’ 5’-GAGCTGGGTCTTTTCAGAATAATAAG-3’

C/EBPα NM_007678 5’-CTGGAAAGAAGGCCACCTC-3’ 5’-AAGAGAAGGAAGCGGTCCA-3’

C/EBPβ NM_009883 5’-TGATGCAATCCGGATCAA-3’ 5’-CACGTGTGTTGCGTCAGTC-3’

C/EBPδ NM_007679 5’-GGGCAGTGGAGTAAGGTACAGA-3’ 5’-GCACTGTCACCCATACAATGTT-3’

aP2 NM_024406 5’-GCCAGACACCCCTGCTA-3’ 5’-GTTCTGGGCGTCACTCC-3’

GLUT4 NM_009204 5’-GACGGACACTCCATCTGTTG-3’ 5’-GCCACGATGGAGACATAGC-3’

ACC NM_133360 5’-GCGTCGGGTAGATCCAGTT-3’ 5’-CTCAGTGGGGCTTAGCTCTG-3’

FAS NM_007988 5’-GTTGGGGGTGTCTTCAACC-3’ 5’-GAAGAGCTCTGGGGTCTGG-3’

SCD NM_009127 5’-TTCCCTCCTGCAAGCTCTAC-3’ 5’-CAGAGCGCTGGTCATGTAGT-3’

SREBP-1c NM_011480 5’-GGTTTTGAACGACATCGAAGA-3’ 5’-CGGGAAGTCACTGTCTTGGT-3’

ATGL NM_001163689 5’-TGACCATCTGCCTTCCAGA-3’ 5’-TGTAGGTGGCGCAAGACA-3’

HSL NM_010719 5’-GCACTGTGACCTGCTTGGT-3’ 5’-CTGGCACCCTCACTCCATA-3’

MGL NM_011844 5’-TCGGAACAAGTCGGAGGT-3’ 5’-TCAGCAGCTGTATGCCAAAG-3’

TBP NM_013684 5’-GTGATGTGAAGTTCCCCATAAGG-3’ 5’-CTACTGAACTGCTGGTGGGTCA-3’

Acc.No. : DDBJ/EMBL/GenBank accession number
